# Transactioos of the Am. Med, Association, Vol, 3

**Published:** 1850-11

**Authors:** 


### ARTICLE IV.

### TRANSACTIONS OF THE AM. MED. ASSOCIATION, VOL. 3.

We have received this work too late for extended notice in this number. It looks well, and from a glance over its contents we have no doubt it will prove to be highly interesting to the profession generally. Though not as large as its predecessor it contains five hundred pages, save one, and is executed in the same excellent style. A notice of its contents may be expected in our next. In the mean time those of our readers, who prefer to obtain the document for themselves, can do so by remitting three dollars, post paid, to the chairman of the Committee on Publication, Dr. Isaac Hays, Philadelphia. The postage on the volume will be 21 cts. if sent by mail.

